# Archives of Dentistry

**Published:** 1884-04

**Authors:** 


### ARCHIVES OF DENTISTRY.

We have received the first number of this journal, the successor of the defunct *Missouri Dental Journal*, published simultaneously in St. Louis, Chicago, and Atlanta, and welcome it to the field of dental literature. We have refrained from saying anything concerning it until it should make its appearance, and now we have not the space to say what we would.

It is an encouraging sign when the best men in the profession, without any hope of pecuniary gain, devote themselves to the task of furnishing proper professional mental pabulum for their fellow practitioners. The list of editors of "Archives" has not yet been announced, but we are assured that it contains the names of some of the most able men of the southwest. The initial number gives promise of an excellence which, there is every reason to believe, will be maintained. It will be published by J. H. Chambers & Co., medical publishers, of St. Louis, Mo.

